# 4186 Single IRB and the CTSI: Liaison Model for the IRB Reliance Process

**DOI:** 10.1017/cts.2020.349

**Published:** 2020-07-29

**Authors:** Christine Sego Caldwell, Amy J. Trullinger, Scott Denne

**Affiliations:** 1 Indiana University School of Medicine; 2 Indiana CTSI, Indiana University School of Medicine

## Abstract

OBJECTIVES/GOALS: Navigating the NIH Single IRB Policy has been challenging for investigators, study teams, and Human Research Protection Programs (HRPP). In response, the Indiana Clinical and Translational Sciences Institute (CTSI) created an innovative Single IRB Project Manager role (sIRB PM), uniquely placed within the Indiana CTSI. METHODS/STUDY POPULATION: The Single IRB Project Manager role was created in 2018 by the Indiana CTSI in response to the NIH Single IRB Policy for Multi-Site Research. The role of the sIRB PM is to serve as a liaison between the Indiana University HRPP, lead site, coordinating center, and participating sites when Indiana University serves as the Single IRB. This model has proven useful to both the IRB and lead site, notably in the following ways:

- **At study start-up**, the sIRB PM can handle complicated communications among sites and the IRB at the same time the lead site is responsible for many other administrative tasks related to start-up. By absorbing the workload of IRB approval for multiple sites, the sIRB PM provides the lead site more capacity to handle other essential tasks.
- The sIRB PM **translates** new terminology and facilitates processes that are new for sites.

RESULTS/ANTICIPATED RESULTS: Early assessment of this program is predominantly positive. The sIRB PM currently supports 24 external sites. In an NIA-funded 13 site study, all sites were added within 9 months of initial IRB approval of the protocol. This role fills a gap that benefits:

- **IRB staff** by allowing them to fulfill their duties of **screening and review** while leaving some of the reliance organization to the sIRBPM.
- **Lead PI** by allowing them to **focus on conducting the research** instead of the many administrative tasks required for single IRB review.
- **Participating sites** by having a **liaison to enter their amendments and reportable events** into an otherwise closed IRB software system.
- **All parties** by having the **sIRB PM manage document organization, storage, and distribution study-wide**.

DISCUSSION/SIGNIFICANCE OF IMPACT: The CTSI sIRB PM role effectively shifts administrative work caused by the sIRB mandate by merging research coordinator experience with regulatory experience while building upon an existing strong relationship with the HRPP. Future focus is on process education, standardizing pricing structure, and ensuring sufficient budget support in grants.

